# DNA damage induces a SAMHD1-mediated block to the infection of macrophages by HIV-1

**DOI:** 10.1038/s41598-018-22432-4

**Published:** 2018-03-07

**Authors:** Paula Jáuregui, Nathaniel R. Landau

**Affiliations:** Department of Microbiology, NYU School of Medicine, Smilow Research Building, Rm. 1003, 550 First Avenue, New York, 10016 USA

## Abstract

Monocyte-derived macrophages (MDMs) are an important target for HIV-1 despite SAMHD1, a myeloid restriction factor for which HIV-1 lacks a counteracting accessory protein. The antiviral activity of SAMHD1 is modulated by phosphorylation of T592 by cyclin-dependent kinases (CDK). We show that treatment of MDMs with neocarzinostatin, a compound that introduces double strand breaks (DBS) in genomic DNA, results in the decrease of phosphorylated SAMHD1, activating its antiviral activity and blocking HIV-1 infection. The effect was specific for DSB as DNA damage induced by UV light irradiation did not affect SAMHD1 phosphorylation and did not block infection. The block to infection was at reverse transcription and was counteracted by Vpx, demonstrating that it was caused by SAMHD1. Neocarzinostatin treatment also activated an innate immune response that induced interferon-stimulated genes but this was not involved in the block to HIV-1 infection, as it was not relieved by an interferon-blocking antibody. In response to Neocarzinostatin-induced DNA damage, the level of the CDK inhibitor p21^cip1^ increased which could account for the decrease of phosphorylated SAMHD1. The results show that the susceptibility of MDMs to HIV-1 infection can be affected by stimuli that alter the phosphorylation state of SAMHD1, one of which is the DNA damage response.

## Introduction

While CD4+ T cells are the major cell-type infected by HIV-1, macrophages play an important role in the replication of the virus *in vivo* and in AIDS pathogenesis. The cells are long-lived and resistant to the cytopathic effects of HIV-1 infection and because of their immunological role in interacting with T cells, have an enhanced propensity to transfer the virus^1^. Monocyte derived macrophages (MDMs) express CD4 and CCR5, rendering them susceptible to virus entry but also express the myeloid restriction factor sterile alpha motif domain and HD domain-containing protein 1 (SAMHD1) that reduces their susceptibility to HIV-1 infection.

The ability of HIV-1 to infect MDMs is limited by SAMHD1, a deoxynucleoside-triphosphate (dNTP) triphosphohydrolase that restricts the replication of HIV-1 in non-cycling monocytes, MDMs, dendritic cells (DCs) and resting T-cells where it depletes the intracellular pool of dNTPs^2–5^, resulting in a block to virus replication at reverse transcription. SAMHD1 also binds single-stranded DNA and RNA^6–10^ and is reported to have DNase and RNase activity^6,11^; however, whether these activities play a role in SAMHD1 antiviral activity is unclear^7–9,12,13^. SIV and HIV-2 encode the accessory protein Vpx that counteracts SAMHD1 by inducing its proteasomal degradation by an association with the CRL4^DCAF1^ E3 ubiquitin ligase complex^14–18^. Degradation of SAMHD1 is accompanied by an increase in the dNTP concentration, allowing the virus to complete reverse transcription. HIV-1 lacks Vpx and has no other means to counteract the restriction^4^.

The antiviral activity of SAMHD1 is regulated by phosphorylation of amino acid T592, which results in the loss of antiviral restriction activity. The mechanism by which T592 phosphorylation regulates SAMHD1 antiviral activity is not fully understood as it has a minimal effect on dNTPase activity. Furthermore, the phosphomimetic mutation T592E lacks antiviral activity yet retains phosphohydrolase activity as measured *in vitro* and depletes dNTP levels in cell-lines^19,20^. In cycling T cells, SAMHD1 is constitutively phosphorylated by cyclin dependent kinase 1 (CDK1) and does not restrict HIV-1 replication^19–21^. In myeloid and resting lymphoid cells, where SAMHD1 exists as a mixture of phosphorylated and dephosphorylated forms, the phosphorylation is mediated by CDK2^22^.

Polymorphisms in the SAMHD1 gene are associated with Aicardi-Goutières Syndrome (AGS), an early onset autoimmune inflammatory disease characterized by constitutive type I interferon (IFN) expression^23,24^. AGS is also associated with mutations in 3′-repair exonuclease 1 (TREX1), ribonuclease H2 (RNASEH2A, RNASEH2B, RNASEH2C), RNA-specific adenosine deaminase 1 (ADAR1) and melanoma differentiation-associated protein 5 (MDA5)^24–28^ all of which are enzymes that play roles in nucleic acid metabolism. The mechanisms by which genetic deficiencies in these proteins causes AGS are not fully understood one possibility is that they are associated with the accumulation of nucleic acids in the cytoplasm that trigger innate immune response sensors, leading to the induction of type I IFN. Such nucleic acids could be the result of genomic DNA damage or endogenous retroviruses^29,30^. SAMHD1 is also mutated in some cases of chronic lymphocytic leukemia^31^ and colorectal cancer^32^, suggesting that it may play a role in genome stability.

In addition to its antiviral role, SAMHD1 is thought to play a role in cell physiology by regulating the intracellular dNTP pool and facilitates DNA repair by homologous recombination (HR) double strand breaks (DSB) repair^4,29,31^. Upon DSB DNA damage, p53 is activated resulting in increased levels of the cyclin dependent kinase inhibitor p21^Cip1^ (p21) which binds to CDKs to inhibit their kinase activity and arrest the cell-cycle. Increased p21 activity is associated with a decrease in phosphorylated SAMHD1, leading to increased antiviral activity^33–35^. p21 also regulates ribonucleotide reductase, an enzyme that increases the intracellular dNTP concentration, which may also influence HIV-1 replication in MDMs^35,36^. Therefore, the DNA damage response could affect SAMHD1 phosphorylation state and thereby affect the susceptibility of MDMs to HIV-1 infection^33,37^.

DNA damage can be induced experimentally using agents that introduce DSBs or that chemically alter the DNA. Neocarzinostatin (NCS), a macromolecular chromoprotein with antitumoral properties, introduces DSBs in genomic DNA. These can be repaired by HR in replicating cells during S/M phase, or by nonhomologous end joining (NHEJ) in non-dividing cells. NCS is active irrespective of the cell cycle and thus should be active in non-cycling MDMs. Camptothecin (CPT), a topoisomerase I inhibitor that induces DSBs is primarily active in the S/M phase of the cell cycle. UV light induces covalent crosslinks between adjacent pyrimidine bases in genomic DNA forming cyclobutane pyrimidine dimers (CPDs). DNA damage induced by UV light is cell-cycle-independent and is repaired through the process of nucleotide excision repair. DNA damage results in the phosphorylation of the variant histone H2AX (γH2AX), which forms foci at sites of DNA damage and contributes to the recruitment of repair proteins to the damaged sites^38,39^. γH2AX serves as an early marker of the DNA damage response. NHEJ repair of DSB induces 53BP1 foci that compete with BRCA1 which is activated in homologous DNA repair^40^. Thus, 53BP1 foci serve as a marker of the DNA damage response in NHEJ.

In this study we explored the role of the DNA damage response in regulating the susceptibility of MDMs to HIV-1 infection. We found that NCS induced a block to the infection of MDMs by HIV-1. UV light damaged MDM DNA but did not induce the block. The block to infection resulted from a decrease in phosphorylated SAMHD1, which was associated with an increase in p21 levels. DNA damage that induced DSBs caused an innate immune response in the MDMs resulting in the release of type-I IFN and the induction of interferon-stimulated genes (ISGs), but these were not the cause of the block to infection. The findings demonstrate that MDM susceptibility to HIV-1 can vary depending on the phosphorylation state of SAMHD1 raising the possibility that it may be possible to regulate the susceptibility of cells to HIV-1 infection by increasing the antiviral activity of SAMHD1.

## Results

### NCS and UV light induce a DNA damage response in MDMs

**Figure 1:**
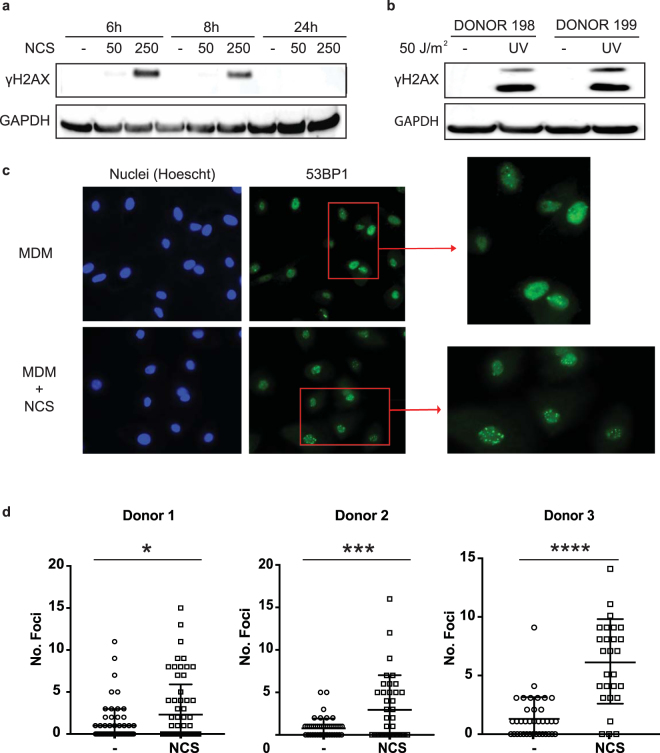
NCS and UV light irradiation induce a DNA damage response in MDMs. NCS induces NHEJ repair in MDMs. (**a**) MDMs were treated with 50 or 250 ng/ml NCS and harvested 6, 8 or 24 h later. The membrane was cut and probed for γH2AX or GAPDH as a loading control. (**b**) MDMs were irradiated with 50 J/m^2^ UV light and lysed after 4 h. The membrane was probed for γH2AX and GAPDH. (**c**) MDMs were treated with 250 ng/ml NCS for 4 h and then fixed and stained with anti-53BP1 antibody (Green) and Hoescht 33342 (Blue). 53BP1 foci were visualized by confocal microscopy. (**d**) 53BP1 foci in the nucleus of each NCS-treated MDM from three donors were quantified (*P-value < 0.05; ***P-value ≤ 0.005; (ns) non-significant, unpaired t-test). MDMs from donors 1 and 2 were treated with 250 ng/ml NCS for 4 h and donor 3 for 6 h. Blots in (**a**) and (**b**) were from samples separated on a single polyacrylamide gel.

To study the effect of DNA damage on the susceptibility of MDMs to HIV-1 infection, we differentiated monocytes of donor PBMCs to MDMs, treated them with NCS, CPT and UV light irradiation, and then detected the DNA damage response by immunoblot analysis for γH2AX phosphorylation. To detect a response to NCS, MDMs were treated with 50 or 250 ng/ml for 6, 8 or 24 h. The results showed that 250 ng/ml NCS generated a DNA damage signal 6 h post-treatment. The response began to resolve by 8 h and was fully resolved by 24 h, reflecting the ability of the MDMs to repair the damage (Fig. 1a). The lower dose did not appear to induce a DNA damage response. To test the effect of UV light, we irradiated MDMs from two donors with 50 J/m^2^. UV light stimulated a strong DNA damage response reflected in the amount of γH2AX (Fig. 1b). CPT did not induce a DNA damage response presumably because MDMs are arrested in G_0_ and was not used further in this study (data not shown).

An early response to DSBs is the formation at the sites of DNA damage in the genomic DNA of nuclear foci that contain 53BP1, an event that is specific to NHEJ repair. To visualize the foci induced in the damaged DNA of MDMs, we treated MDMs from three donors with NCS for 4 h and then quantified the 53BP1 foci (Fig. 1d). In the absence of the drug, a small number of foci were visible in the nuclei of some of the cells, reflecting a low level of ongoing DNA damage and repair. Upon treatment with NCS, the number of foci significantly increased (Fig. 1c and d), a result that was consistent in MDMs from three donors. We concluded that NCS and UV light damaged the DNA of MDMs and induced a response to repair the damage.

### Generation of DSBs in MDM genomic DNA induces ISGs

**Figure 2:**
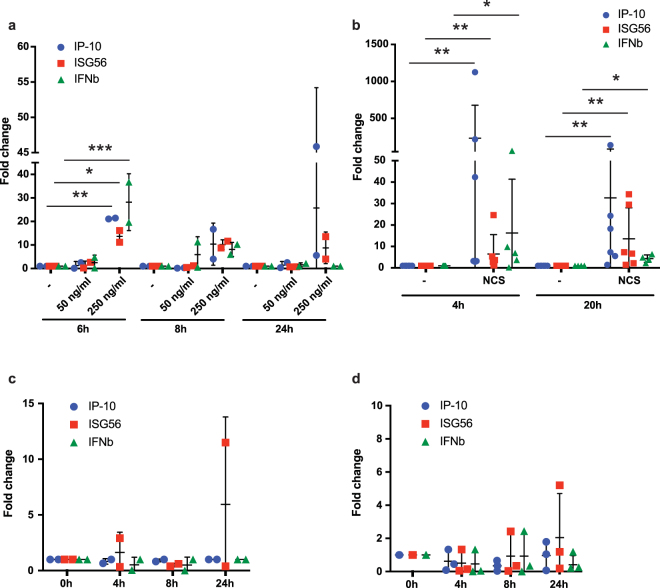
The DNA damage response to NCS but not UV light induces type I IFN in MDMs. (**a**) MDMs were treated with 50 or 250 ng/ml NCS. After 6, 8 or 24 h RNA was prepared and IP-10, ISG56 and IFNβ mRNA transcripts were quantified by RT-qPCR. The ΔΔCT relative to GAPDH in the untreated samples was set to 1 (n = 2; *P-value < 0.05; **P-value ≤ 0.01; ***P-value ≤ 0.001; no symbol indicates non-significant by two-way ANOVA test). Each dot represents one donor. Comparisons made on untreated vs treated for each time point. (**b**) MDMs were treated for 4 h with 250 ng/ml NCS and then harvested or incubated without drug for 16 h. RNA was prepared and IP-10, ISG56 and IFNβ mRNA transcripts were quantified by RT-qPCR. (n = 6; *P-value < 0.05; **P-value ≤ 0.01; no symbol indicates not significant by Kolmogorov-Smirnov test). (**c**) MDMs were irradiated with 50 J/m² UV light and harvested at 0, 4, 8 or 24 h. IP-10, ISG56 and IFNβ mRNA transcripts were quantified by RT-qPCR. (n = 2; no symbol indicates not significant by 2-way ANOVA and Kruskal-Wallis tests). Comparisons made with 0 h time point. (**d**) MDM were irradiated with 10 J/m² UV light and analyzed as in (**c**). (n = 3; no symbol indicates not significant by 2-way ANOVA and Kruskal-Wallis tests). Comparisons made with 0 h time point.

To determine whether DNA damage induces ISGs in MDMs, we treated MDMs with NCS or UV irradiation and then quantified the induction of interferon β (IFNβ) and the ISGs IP-10 and ISG56. To measure ISG induction in response to DNA damage, we treated MDMs with 50 or 250 ng/ml NCS and after 6, 8 and 24 h quantified mRNA transcripts for IFNβ, IP-10 and ISG56 by RT-qPCR. 50 ng/ml NCS had no effect on ISG expression (Fig. 2a), consistent with the lack of DNA damage response at this concentration. 250 ng/ml NCS, which did induce a DNA damage response, at 6 h post-treatment, induced a 20-fold increase in IP-10 and IFNβ mRNA transcripts and 15-fold increase in ISG56 (Fig. 2a). These gradually decreased at 8 and 24 h becoming insignificant, a time course similar to that for the induction of γH2AX. To determine how quickly the response occurs and how long it lasts, we measured the response after 4 h NCS treatment and after a 16 h recovery period. After 4 h, IFNβ, IP-10 and ISG56 transcripts increased 15-fold, 200-fold and 6-fold, respectively (Fig. 2b). After the 16 h recovery, the number of IFNβ transcripts had nearly returned to baseline while IP-10 and ISG56 remained somewhat elevated.

To measure the innate immune response to UV light, we irradiated MDMs with 50 or 10 J/m^2^ UV light and at 0, 4, 8 or 24 h quantified the ISG mRNA transcripts. UV irradiation did not induce an IFN response (Fig. 2c and d). This suggests that DSBs induce an innate immune response while the introduction of CPDs does not. This is a surprising difference as both treatments provoked a strong DNA damage response as reflected by γH2AX detection.

### DNA damage leads to a decrease of phosphorylated SAMHD1 and a block to HIV-1 infection of MDMs

**Figure 3:**
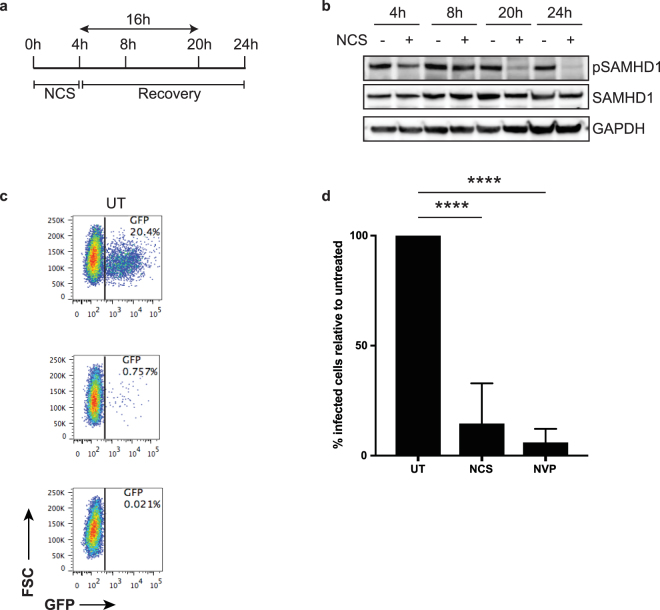
NCS treatment of MDMs leads to lower amounts of phosphorylated SAMHD1 and a block to HIV-1 infection. (**a**) A timeline for the course of NCS treatment and cell harvest is shown. MDMs were treated for 4 h with NCS. Then cells were processed or allowed to recover for 16 h in the absence of drug. As controls, cells were untreated. (**b**) MDMs were treated for 4 h with 250 ng/ml NCS. The cells were incubated for 4, 16 or 20 h longer without drug and then harvested and lysed. The samples were separated on two different polyacrylamide gels. One membrane was probed for pSAMHD1. The other was cut and probed for SAMHD1 and GAPDH. (**c**) Flow cytometry plots of untreated MDM (UT) or MDM treated with 250 ng/ml NCS for 4 h + 16 h without drug. After 20 h, the cells were infected with HIV-1.GFP. Three days later, the MDMs were analyzed by flow cytometry. MDMs were treated with NVP as a negative control. (**d**) The percentage of infected MDMs relative to untreated MDMs is shown for five donors treated as in (**b**). Untreated MDMs were set to 100%. (n = 5; ****P-value < 0.0005; Mann-Whitney test). Comparisons are with untreated cells.

The myeloid cell restriction factor SAMHD1 is expressed in MDMs but exerts only a modest effect on HIV-1 replication in the cells, most likely because it is largely in the phosphorylated, inactive state. Because of the role of SAMHD1 in DNA damage repair, we tested whether DNA damage would affect the phosphorylation state of SAMHD1. To do this, we treated MDMs with NCS for 4 h and then removed the drug (Fig. 3a). We then harvested the cells at 4, 8, 20 or 24 h and detected total and phospho-SAMHD1 (pSAMHD1) by immunoblot analysis. At 20 and 24 h post-treatment there was a significant decrease in the amount of pSAMHD1 while the total amount of SAMHD1 remained unchanged (Fig. 3b).

Because SAMHD1 dephosphorylation activates its restriction activity, this result predicted that NCS treatment would decrease the susceptibility of MDMs to HIV-1 infection. To test this possibility, we treated MDMs with NCS for 4 h and after 16 h of recovery infected them with HIV.GFP. As predicted, the MDMs became resistant to HIV-1 infection (Fig. 3c and d and Suppl. Figure 1a). In this experiment, we used donor MDMs with above average infectabilty with Vpx− virus to better illustrate the effect. The results were confirmed with four additional donors (Fig. 3d; raw data shown in Suppl. Figure 1a). Treatment of the cells with the reverse transcriptase inhibitor nevirapine (NVP) confirmed that the GFP + cells were the result of productive infection. These results suggest that DNA damage that causes DSBs activated the restriction activity of SAMHD1, blocking infection by HIV-1.

### Vpx overcomes the DNA damage-induced block to HIV-1 infection

**Figure 4:**
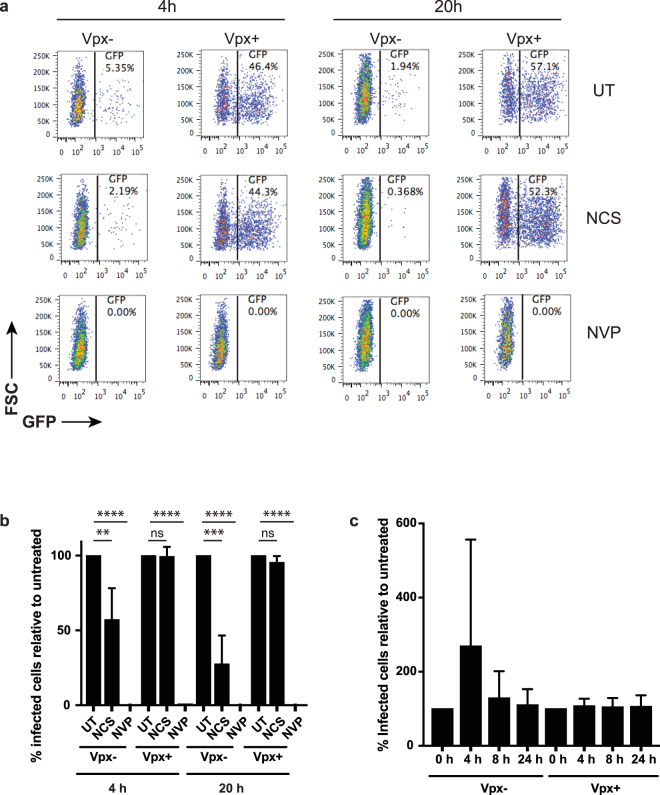
Vpx overcomes the DNA damage-induced block to HIV-1 infection. (**a**) Flow cytometry plots of MDMs treated for 4 h with 250 ng/ml NCS or untreated (UT) and then infected with HIV-1.GFP with (Vpx+) or without (Vpx−) Vpx or incubated for 16 h longer without drug and then infected 20 h after treatment with HIV-1.GFP Vpx+ or Vpx−. Three days later, the MDMs were analyzed by flow cytometry. MDMs were treated with NVP as a negative control. (**b**) The percentage of GFP + MDMs (infected cells) relative to untreated MDMs from three donors treated as in (**a**). Untreated MDMs were set to 100%. (n = 3; **P-value < 0.01; ***P-value < 0.001; ****P-value < 0.0005; one-way ANOVA test). (**c**) MDMs were irradiated with 10 J/m² UV light and then infected immediately or incubated 4, 8 or 24 h and infected with HIV-1.GFP Vpx + or Vpx−. Three days later, the cells were analyzed by flow cytometry. The percentage of GFP + MDMs (infected cells) relative to untreated MDMs from two donors. Untreated MDMs were set to 100%. (n = 2; no symbol indicates not significant by Kruskal-Wallis test).

NCS treatment was associated with a decrease in phosphorylated SAMHD1 and ISG expression, either or both of which could have been responsible for the block to HIV-1 infection. To determine whether the lower amount of phosphorylated SAMHD1 was responsible for the block to infection induced by NCS, we tested whether Vpx, which degrades SAMHD1, would alleviate the inhibition of infection using HIV-1 virions containing Vpx. The Vpx-containing virions were produced as previously reported by co-transfection of 293 T cells with an HIV-1 provirus modified to contain the Vpx packaging motif in Gag p6 and an SIV_mac239_ Vpx expression vector^41^. We treated MDMs with NCS for 4 h, allowed them to recover for 16 h and then infected them with Vpx-containing (Vpx+) or lacking (Vpx−) HIV.GFP. In the untreated MDMs, Vpx− virus infected the cells to a low level (Fig. 4a and b and Suppl. Figure 1b). NCS treatment further reduced the infection frequency. In untreated MDMs, Vpx+ virus infected with much higher infectivity and NCS treated MDMs were infected with similar efficiency (Fig. 4a and b and Suppl. Figure 1b). Treatment of the MDMs with NVP confirmed that the GFP+ cells were the result of productive infection.

To test whether UV light induced DNA damage would also cause a SAMHD1-mediated block to HIV-1 infection, we repeated the analysis substituting NCS treatment with UV irradiation. We irradiated MDMs with 10 J/m^2^ UV, a dose that allowed the MDMs to remain viable over the time course of the experiment, and then infected the cells with Vpx+ or Vpx− HIV.GFP at 0, 4, 8 or 24 h. There was no difference in infection between cells infected at 0, 4, 8 or 24 h (Fig. 4c and Suppl. Figure 1c). The Vpx effect was the same at every time point. We concluded that the block to infection induced by NCS is caused by SAMHD1. The block to infection was specific for damage that induced DSBs as UV light irradiation did not induce the block.

### DNA damage-induced decrease of phosphorylated SAMHD1 is associated with increased p21 and is type-I IFN independent

**Figure 5:**
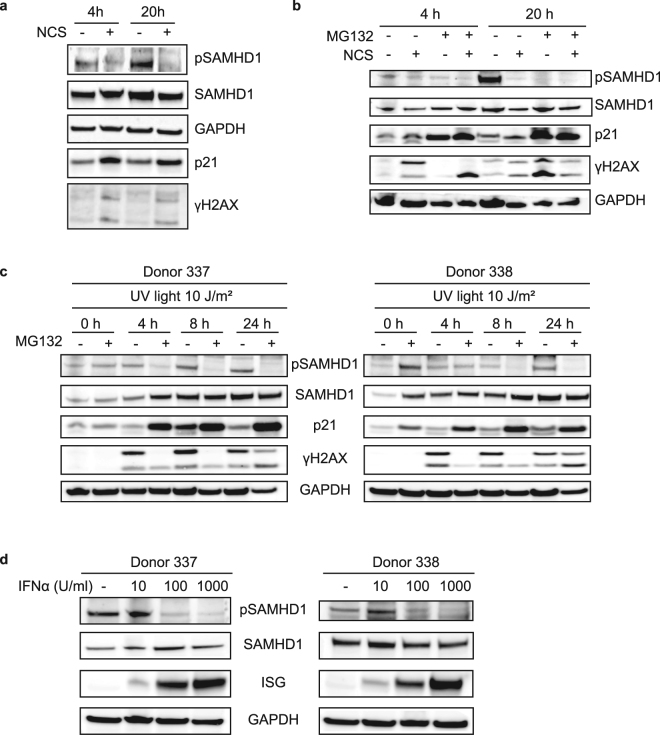
DSB DNA damage-induced decrease of pSAMHD1 is associated with an increase in p21 level and is not the result of type I IFN. (**a**) MDMs were treated for 4 h with NCS. The drug was removed and the MDMs were harvested or incubated for 16 h. Lysates were prepared and analyzed on two immunoblots. One membrane was cut and probed for pSAMHD1 and γH2AX. The other was cut and probed for SAMHD1 and p21. The membrane probed for p21 was stripped and then probed for GAPDH. (**b**) MDMs were treated with NCS for 4 h and incubated with MG132. After 4 h, NCS was removed and the MDMs were harvested or incubated for 16 h in presence of MG132. Lysates were prepared and analyzed on two immunoblots. One membrane was cut and probed for pSAMHD1 and γH2AX. The other was cut and probed for SAMHD1 and p21. The membrane probed for p21 was stripped and then probed for GAPDH as a loading control. (**c**) MDMs were irradiated with 10 J/m^2^ UV light and incubated or not with MG132. The cells were lysed immediately (0 h) or incubated for 4, 8 or 24 h. The lysates were analyzed on immunoblots as in (**c**). Two donors are shown. (**d**) MDMs were untreated or treated with increasing amounts of IFNα for 20 h. The lysates were separated on two gels for immunoblot analysis. One membrane was probed for pSAMHD1 and viperin as a control ISG. The other was cut and probed for SAMHD1 and GAPDH. Two donors are shown.

Because NCS and UV light activate different pathways for DNA repair, we wanted to know whether both induce the decrease of phosphorylated SAMHD1 levels. SAMHD1 is phosphorylated in MDMs by CDK1/2, both of which are inhibited by p21. To determine the effect of DNA damage on p21 levels, we treated MDMs with NCS for 4 h and lysed them immediately or after 16 h in the absence of the drug. We then measured the amount of total SAMHD1, pSAMHD1, p21 and γH2AX by immunoblot analysis. The results showed that NCS treatment caused an increase in the amount of p21 at 4 and 20 h that was associated with a decrease of the amount of pSAMHD1 and an increase in γH2AX (Fig. 5a).

To test whether the decreased amount of pSAMHD1 was due to degradation of the phosphorylated form of SAMHD1, we treated MDMs with NCS in the presence of the proteasome inhibitor MG132. The MDMs were then lysed immediately or after 16 h. We then analyzed total SAMHD1, pSAMHD1, p21 and γH2AX by immunoblot analysis. The results showed that the decrease of pSAMHD1 levels upon NCS treatment was not affected by MG132, suggesting that it was not the result of proteasomal degradation of pSAMHD1. Interestingly, MG132 treatment led to an increase in the level of p21 and a decrease in pSAMHD1 (Fig. 5b). These results suggest a model in which NCS induces p21, which inhibits CDK1/2 phosphorylation of SAMHD1.

To test whether UV light irradiation was associated with a decrease in pSAMHD1 levels and increased p21, we irradiated MDMs with 10 J/m^2^ UV in the presence or absence of 10 μM MG132 and after 0, 4, 8 or 24 h determined the levels of SAMHD1 and p21 by immunoblot analysis. UV irradiation did not cause the decrease of pSAMHD1 amount (Fig. 5c). The DNA damage response was confirmed by the increase in γH2AX (Fig. 5c). MG132 treatment caused an increase in p21 and the decrease of pSAMHD1 (Fig. 5c), further suggesting that the decrease of pSAMHD1 levels is caused by p21.

Because DNA damage is associated with the induction of ISGs including type-I IFN, it is possible that the decrease of pSAMHD1 induced by DNA damage was caused by type-I IFN. To test whether type-I IFN itself induced the decrease in pSAMHD1, we treated MDMs with increasing doses of IFNα for 20 h and measured the amount of total and pSAMHD1 by immunoblot analysis. The results show that IFNα reduced pSAMHD1 levels, consistent the results from Cribier *et al*.^21^. To determine whether IFN is responsible for the lower amount of pSAMHD1 upon NCS treatment, we treated MDMs with NCS for 4 h in the presence of a blocking concentration of anti-IFN receptor (IFNAR) antibody^42^. The MDMs were lysed after 4 h or 16 h and total SAMHD1, pSAMHD1 and γH2AX was analyzed on an immunoblot. The results showed no effect of the anti-IFNAR antibody on pSAMHD1 levels (Suppl. Figure 2a). We concluded that type-I IFN production is not required for the decrease in pSAMHD1.

### The block to infection mediated by SAMHD1 is at early reverse transcription

**Figure 6:**
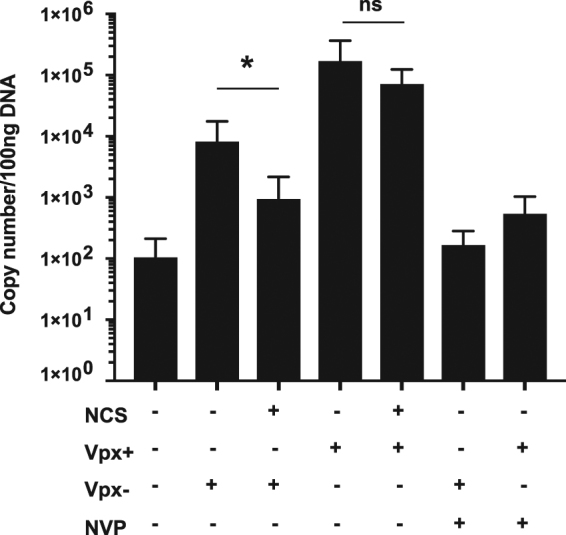
The DNA damage-induced block to HIV-1 infection is at early reverse transcription. MDMs were treated for 4 h with 250 ng/ml NCS and then incubated 16 h without drug. The MDMs were then infected with Vpx + or Vpx− DNase-treated luciferase reporter virus. 48 h later, DNA was prepared and early reverse transcription products were quantified by qPCR in the cells of three donors. MDMs were treated with NVP to control for contaminating HIV-1 plasmid DNA. (n = 3; *P-value < 0.05; ns indicates not significant by Wilkoxon test).

To determine whether DNA damage results in inhibition of HIV-1 infection at reverse transcription, we quantified HIV-1 DNA synthesized after infection of NCS-treated MDMs. To increase the sensitivity of the analysis, we cultured the MDMs in medium containing FBS rather than PHS, which increases the infectability of MDMs to HIV-1 infection^37^ allowing for a more robust analysis. For this analysis, we infected the cells with luciferase reporter virus, a virus that is similarly affected by NCS treatment (Suppl. Figure 3). We treated MDMs with NCS for 4 h, incubated 16 h without drug, and then infected with DNAse-treated Vpx− or Vpx+ HIV-1. After 48 h, we quantified newly synthesized viral DNA molecules by qPCR using primers that detect the early products of reverse transcription. NVP was added to control for plasmid DNA contamination. The infection of NCS-treated MDMs with Vpx− virus resulted in the synthesis of more than 10-fold less viral DNA molecules in three donors (Fig. 6). Infection of the treated cells with Vpx+ virus increased viral DNA synthesis in the three donors compared to cells infected with Vpx− and there was no difference in Vpx+ infection between MDMs treated with NCS or not. The results suggest that the NCS-induced block to HIV-infection is at early reverse transcription, consistent with SAMHD1-mediated restriction.

## Discussion

MDMs are an important target cell-type for HIV-1 *in vivo* despite their expression of SAMHD1, a restriction factor for which the virus lacks a counteracting accessory protein. Moreover, MDMs are non-dividing cells, a feature that generally enhances SAMHD1 antiviral activity. We show here that the ability of HIV-1 to infect MDMs results from the partial inactivation of SAMHD1 as a result of phosphorylation and that treatments that alter the phosphorylation state of SAMHD1 render the cells resistant to infection. The treatment of MDMs with NCS to introduce DSBs, or by irradiation with UV light to introduce CPDs in the genomic DNA, induced a DNA damage response as detected by H2AX phosphorylation. NCS treatment, but not UV irradiation, induced a block to HIV-1 infection in the MDMs that resulted from the decrease of phosphorylated SAMHD1 levels. The block to infection was at early reverse transcription and was overcome by packaging Vpx into the virions, demonstrating that the block was caused by SAMHD1 and not by other cellular alterations induced by DNA damage. The DNA damage response was accompanied by an increase in the level of p21, which inhibits the CDKs that phosphorylate SAMHD1 and may thus account for the decrease of the phosphorylated form of SAMHD1.

The production of DSBs induced by NCS treatment caused the induction of IFNβ and the ISGs IP-10 and ISG56. In contrast, UV light, which causes the formation of covalent linkages in adjacent purine bases in genomic DNA, had little effect on IFNβ or ISG expression although it provoked a strong DNA damage response as measured by the activation of γH2AX. A potential explanation for this difference is that DSBs, as opposed to cross-linked bases, may lead to the accumulation of cytoplasmic DNA. Cytoplasmic DNA activates cGAS to synthesize cGAMP, which through signaling by STING, triggers the induction of ISGs including type-I IFN, and it has been shown that DNA damage resulting in cytoplasmic DNA activates this pathway^43–45^. UV light does not typically induce DSBs in non-dividing cells that would result in cytoplasmic DNA and does not activate IRF3, a transcription factor involved in the induction of ISGs^46,47^. While the ISGs induced by DSBs did not prevent the infection of MDM in a single round of replication, they could cause an antiviral state in bystander cells that would block subsequent rounds of HIV-1 replication. Furthermore, the activation of the IFN pathway upon DBS could be the reason for the excessive IFN production in AGS patients. Since SAMHD1 is inactivated in AGS, the cells might not be able to repair DBS. Thus damaged DNA can go into the cytoplasm and be accessible to DNA sensors that activate the IFN pathway constantly.

Several models could account for the decreased amount of pSAMHD1 upon DNA damage. NCS increased levels of the CDK1/2 inhibitor p21. As SAMHD1 is a substrate for these kinases^33,48^, their inhibition could account for the decrease in phosphorylated SAMHD1. Moreover, the treatment of MDMs with MG132 caused an increase in the level of p21 and a decrease in pSAMHD1 levels, supporting the idea that p21 is involved in the reduction of pSAMHD1 amount. Alternatively, NCS could activate a phosphatase for which SAMHD1 is a substrate. One candidate phosphatase is PP2A; however, PP2A levels were not affected by NCS treatment (data not shown). It was also possible that NCS induces the proteasomal degradation of phosphorylated SAMHD1. Arguing against this possibility, MG132 did not prevent the NCS-induced loss of phosphorylated SAMHD1. Another possible mechanism was that the decrease in pSAMHD1 amount was caused by type I IFN induced by NCS^21^. However, anti-IFNAR antibody did not block the decrease in pSAMHD1 levels.

During the preparation of this manuscript, Mlcochova *et al*. reported that the topoisomerase inhibitor etoposide (ETO) leads to SAMHD1 dephosphorylation, blocks HIV-1 infection in MDMs and is rescued by SAMHD1 depletion^49^, consistent with our findings with damage induced by NCS. Their findings differed from ours in that the block to infection was at nuclear import, not reverse transcription, a difference that could be caused by the use of different DNA damage agents or differences in MDM culture conditions (GM-CSF rather than M-CSF and PHS rather than FBS).

Aside from its role as a viral restriction factor, the physiological role of SAMHD1 has been the subject of several recent reports^30–32,50,51^. As a dNTPase, it regulates the intracellular pool of dNTPs, serving as a means to preserve the quality of the pool by recycling dNTPs that become oxidized or otherwise modified. The activation of SAMHD1 upon DNA damage would intuitively appear to be unfavorable, as it would result in the depletion of dNTPs required for the repair of damaged DNA. However, it is possible that the cell depletes its dNTPs upon DNA damage to prevent unwanted DNA synthesis while the cell is arrested in the cell cycle. Alternatively, Daddacha *et al*. recently showed that SAMHD1 plays a role in HR DNA repair that involves the formation of SAMHD1 complexes at sites of DSBs through an interaction with CtIP. This function is dNTPase-independent and does not involve the catalytic site of the enzyme^29^. Although there is no evidence for SAMHD1 participation in NHEJ DSB repair in non-dividing cells, it is possible that phosphorylation regulates this function of SAMHD1, allowing it when dephosphorylated to form complexes with DNA damage repair proteins. Such a role could provide a rationale for the regulation of SAMHD1 by phosphorylation, which has only a modest effect on its dNTPase activity *in vitro*. It is also possible that the decrease in phosphorylated SAMHD1 induced by DNA damage could affect the replication of viruses that replicate through a DNA intermediate other than HIV-1. For example, vaccinia virus and Herpes Simplex Virus I are restricted by SAMHD1 and murine γ-herpes virus replication is blocked by DNA damage^52–54^.

The phosphorylation state of SAMHD1 appears to be affected by several intracellular factors. Growth of MDMs under conditions that alter the cell cycle, including culturing the cells in medium containing FBS or PHS^37^ or with M-CSF or GM-CSF^33^ alters SAMHD1 phosphorylation and affects their susceptibility to infection by HIV-1. *In vivo*, MDMs are differentiated as M1, M2 or alternatively activated by M-CSF or GM-CSF in the local environment (Reviewed in ref.^55^). Because MDMs play a role in HIV-1 replication, alterations in SAMHD1 restriction activity could influence HIV-1 replication. While DNA damage agents are not likely to be useful in the treatment of HIV-1 infection, it may be possible to control the SAMHD1 phosphorylation state by targeting intracellular signal transduction in MDMs, thereby reducing virus loads.

## Materials and Methods

### Cell culture

293 T cells were cultured in Dulbecco’s Modified Eagle Medium (DMEM) supplemented with 10% fetal bovine serum (FBS) and penicillin/streptomycin. PBMCs were prepared by Ficoll density gradient centrifugation of blood from anonymous donors provided by the New York Blood Center. The monocytes were purified by plastic adherence and cultured in RPMI containing 10 mM HEPES, 24 µg/ml gentamicin, and 5% heat inactivated pooled human serum (PHS) or fetal bovine serum (FBS). MDMs were prepared by culturing the monocytes for 6 days in medium containing 50 ng/ml GM-CSF, which was replenished every two days.

### Virus preparation

To produce Vpx-containing HIV-1 GFP reporter virus (HIV.GFP), 293 T cells were cotransfected by calcium phosphate coprecipitation with HIV.GFP containing the p6 Vpx-packaging motif of SIVmac^41^, pcVSV-G^56^ and pcVpx.mycHis^41^ or pcDNA6 (Invitrogen) at a mass ratio of 10:1:1. Vpx-containing HIV-1 luciferase reporter virus was produced by co-transfecting 293 T cells with the luciferase reporter virus containing the Vpx packaging motif in Gag P6 pNL.luc3.p6* E-R-^41^, pcVSV-G^56^ and pcVpx^41^ or pcDNA6 (Invitrogen) at a mass ratio of 10:1:1.

### Immunoblot analysis

Cells were solubilized in RIPA buffer with 2% SDS. Cell lysates (30μg) were separated by SDS-PAGE, transferred to PVDF membranes and probed. The antibodies used were anti-γH2AX mAb (Millipore), rabbit anti-phospho-SAMHD1 antibody (Cell Signaling Technologies), anti-SAMHD1 mAb (Origene), anti-GAPDH mouse mAb (Ambion), anti-p21 rabbit mAb (Cell Signaling Technologies) and anti-Viperin mAb (Millipore). The membranes were washed and probed with goat anti-mouse or anti-rabbit horseradish peroxidase (HRP)-conjugated second antibody (Sigma) and visualized using HRP substrate (Pierce) on an Odyssey Fc dual-mode imaging system (Li-Cor).

### Confocal microscopy

CD14+ cells were isolated by negative selection from PBMCs using the Dynabead Untouched Human Monocytes kit (Invitrogen). 5 × 10^5^ CD14 + monocytes were plated on poly-L-Lysine treated coverslips and differentiated to MDM for six days. The cells were fixed in 4% paraformaldehyde, blocked and incubated with rabbit anti-53BP1 antibody (Novus) followed by goat anti-rabbit alexa-fluor 488 second antibody. The nuclei were stained with Hoechst 333429 and the cells were visualized with a Leica SP5 confocal microscope and analyzed using ImageJ software.

### qPCR quantification of HIV-1 reverse transcripts

Monocytes were isolated by plastic adherence from 2 × 10^7^ PBMC plated in 6-well plates. The cells were treated with 250 ng/ml NCS for 4 h after which the drug was removed. The cells were then incubated for another 16 h and infected with DNase I/benzonase-treated HIV-1 luciferase reporter virus (2 × 10^8^ cps). As a control, the cells were treated with 10 μM nevirapine. At 48 h post-infection, total DNA was isolated and analyzed by qRT-PCR using SYBR green (Molecular Probes) and primers that amplified early HIV-1 reverse transcripts (early RT: fw 5′-GCT AAC TAG GGA ACC CAC TGC TT-3′ and rev 5′-ACA ACA GAC GGG CAC ACA CTA C-3′). The data were normalized to 100 ng DNA and quantified using a standard curve generated with proviral plasmid DNA serially diluted in 293 T cell genomic DNA.

### Reverse transcriptase qRT-PCR mRNA quantification

RNA was isolated from 2 × 10^7^ monocytes using Trizol and treated with RNase-free DNase I (Roche). cDNA was synthesized with an oligo-dT primer and Transcriptor RT (Roche). cDNA corresponding to 50 ng of RNA was analyzed by qRT-PCR with SYBR green quantification. Primers used for transcripts for IFNβ (fw 5′-AGG ACA GGA TGA ACT TTG AC-3′ and rev 5′-TGA TAG ACA TTA GCC AGG AG-3′), IP-10 (fw 5′-TGC CAT TCT GAT TTG CTG CC-3′ and rev 5′-TGC AGG TAC AGC GTA CAG TT-3′), ISG56 (fw 5′- GGC TAC AAA AGG GCA GCC TA-3′ and rev 5′-GCC AGG TCT AGA TGA GCC AC-3′) and GAPDH (fw 5′-TGG AAG GAC TCA TGA CCA CAG and rev 5′-CAG TCT TCT GGG TGG CAG TGA)^42^. Reactions without RT were included when making cDNA to control for genomic DNA contamination. The ΔΔCT relative to GAPDH in the untreated samples was set to 1.

## Electronic supplementary material

Supplementary Information
