# Supplementary material for: DNA damage induces a SAMHD1-mediated block to the infection of macrophages by HIV-1
Source: Sci Rep. 2018 Mar 7;8:4153. doi: 10.1038/s41598-018-22432-4 (PMC5841438; doi:10.1038/s41598-018-22432-4)
Supplement: Supplementary file 1 — Supplementary Information [file 41598_2018_22432_MOESM1_ESM.pdf]

Supplemental Information

DNA damage induces a SAMHD1-mediated block to the infection of macrophages by HIV-1

Paula Jáuregui and Nathaniel R. Landau[#]

Supplementary Figure Legends.

Supplementary Figure 1. Raw data for figures 3 and 4. a) MDMs were treated with 250 ng/ml NCS for 4h and 16h without drug. After 20h, the cells were infected with HIV-1.GFP. 3 days later, the MDMs were analyzed by flow cytometry. MDMs were treated with NVP as a negative control. The percentage of infected MDMs is represented (n=5; **P-value <0.01; Mann-Whitney test). **b)** MDMs were treated with 250 ng/ml NCS for 4h or untreated (UT) and then infected with HIV-1.GFP with (Vpx+) or without (Vpx-) Vpx or incubated for 16h longer without drug and then infected 20h after the treatment with HIV-1. GFP Vpx+ or Vpx-. 3 days later, the MDMs were analyzed by flow cytometry. The percentage of infected MDMs from 3 healthy donors is represented. **c)** MDMs were irradiated with 10 J/m² UV light and then infected immediately or incubated 4, 8 or 24 h and infected with HIV-1.GFP Vpx+ or Vpx-. 3 days later, the cells were analyzed by flow cytometry. The percentage of GFP+ MDMs from two donors is represented (n=2; no symbol indicates not significant by the Kruskal-Wallis test).

Supplementary Figure 2. The decrease of phosphorylated SAMHD1 levels upon DNA damage is independent of IFN. MDMs were treated with NCS for 4h and incubated or not with 0.5 ng/µl IFNAR antibody. After 4h, NCS was removed and the MDMs were harvested or incubated for 16h in presence or not of IFNAR antibody. Lysates were prepared and analyzed on two immunoblots. One membrane was cut and probed for pSAMHD1 and γH2AX. The other was cut and probed for SAMHD1 and GAPDH as a loading control.

Supplementary Figure 3. The DNA damage-induced block to HIV-1 infection of MDMs using luciferase reporter virus. MDMs were treated with 250 ng/ml NCS for 4h and then incubated for 16h without drug. The MDMs were infected with Vpx+ or Vpx luciferase reporter virus. 72h later, luciferase activity was measured (n=3; **P-value < 0.01; ***P-value < 0.001; ns indicates not significant by the unpaired t-test).

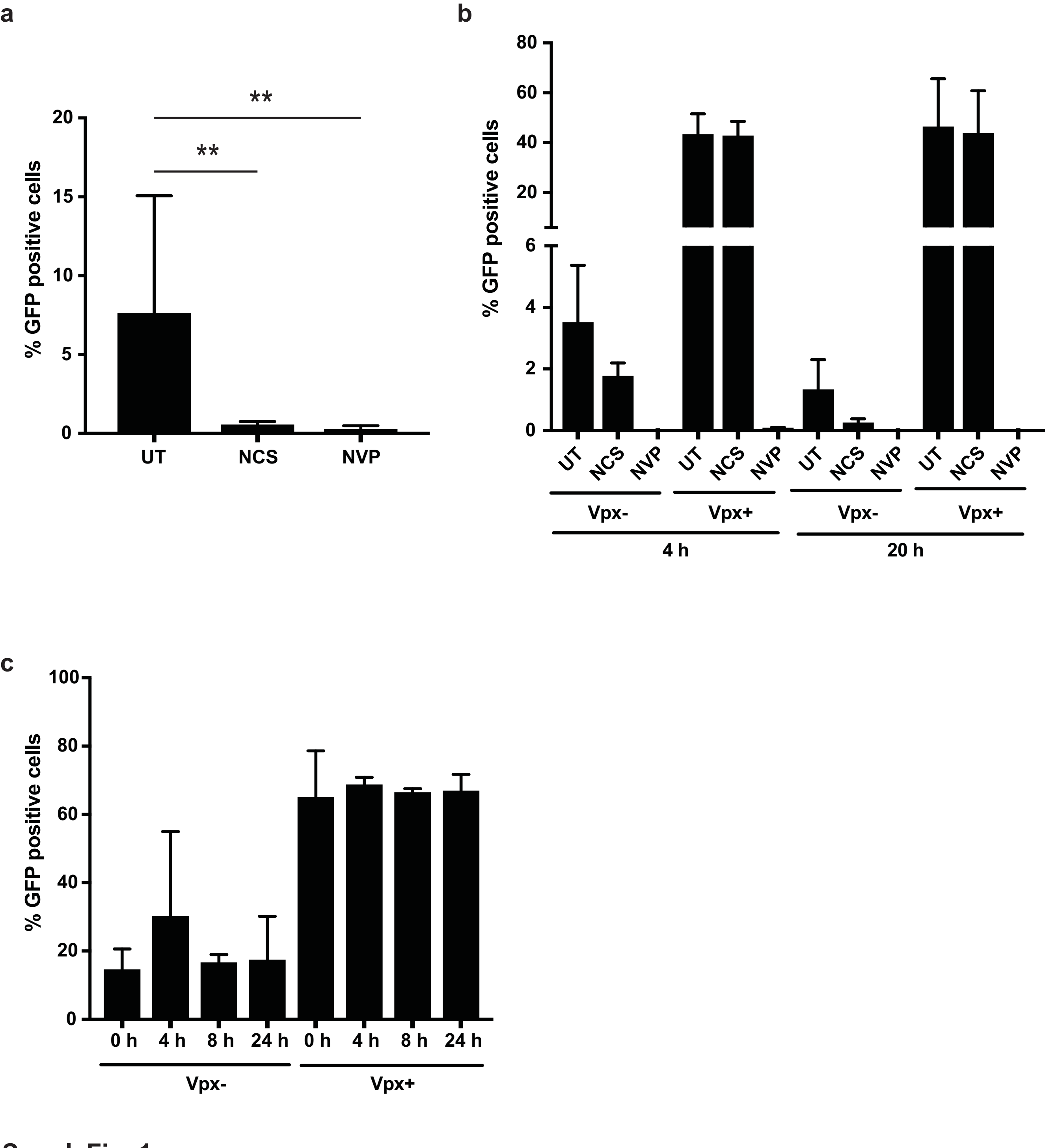
a
% GFP positive cells
0
5
10
15
20
**
**
UT
NCS
NVP
b
% GFP positive cells
0
2
4
6
20
40
60
80
UT
NCS
NVP
UT
NCS
NVP
UT
NCS
NVP
UT
NCS
NVP
Vpx-
Vpx+
Vpx-
Vpx+
4 h
20 h
c
% GFP positive cells
0
20
40
60
80
100
0 h
4 h
8 h
24 h
0 h
4 h
8 h
24 h
Vpx-
Vpx+

Suppl. Fig. 1.

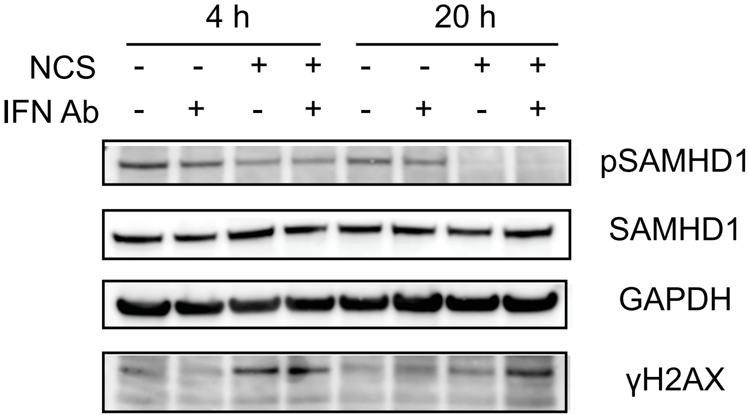

Suppl. Fig. 2.

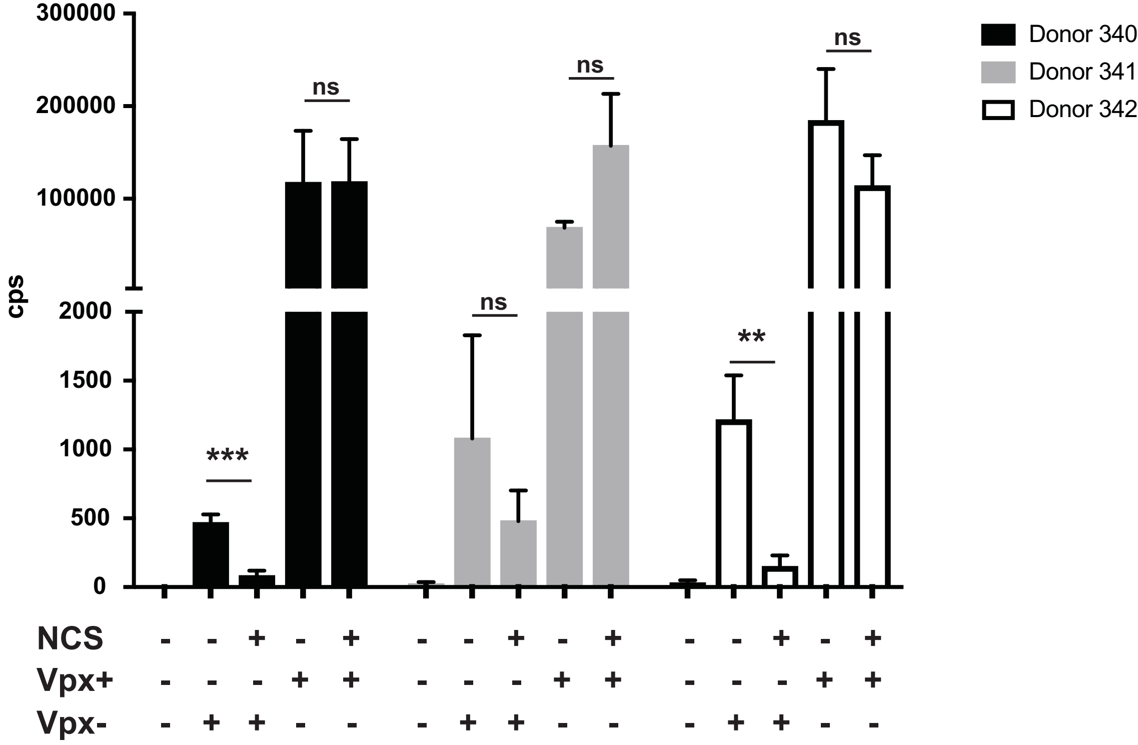

Donor 340
Donor 341
Donor 342
cps
300000
200000
100000
2000
1500
1000
500
0
ns
ns
ns
ns
***
**
NCS - - + - + - - + - + - - + - +
Vpx+ - - - + + - - - + + - - - + +
Vpx- - + + - - - + + - - - + + - -

Suppl. Fig. 3.

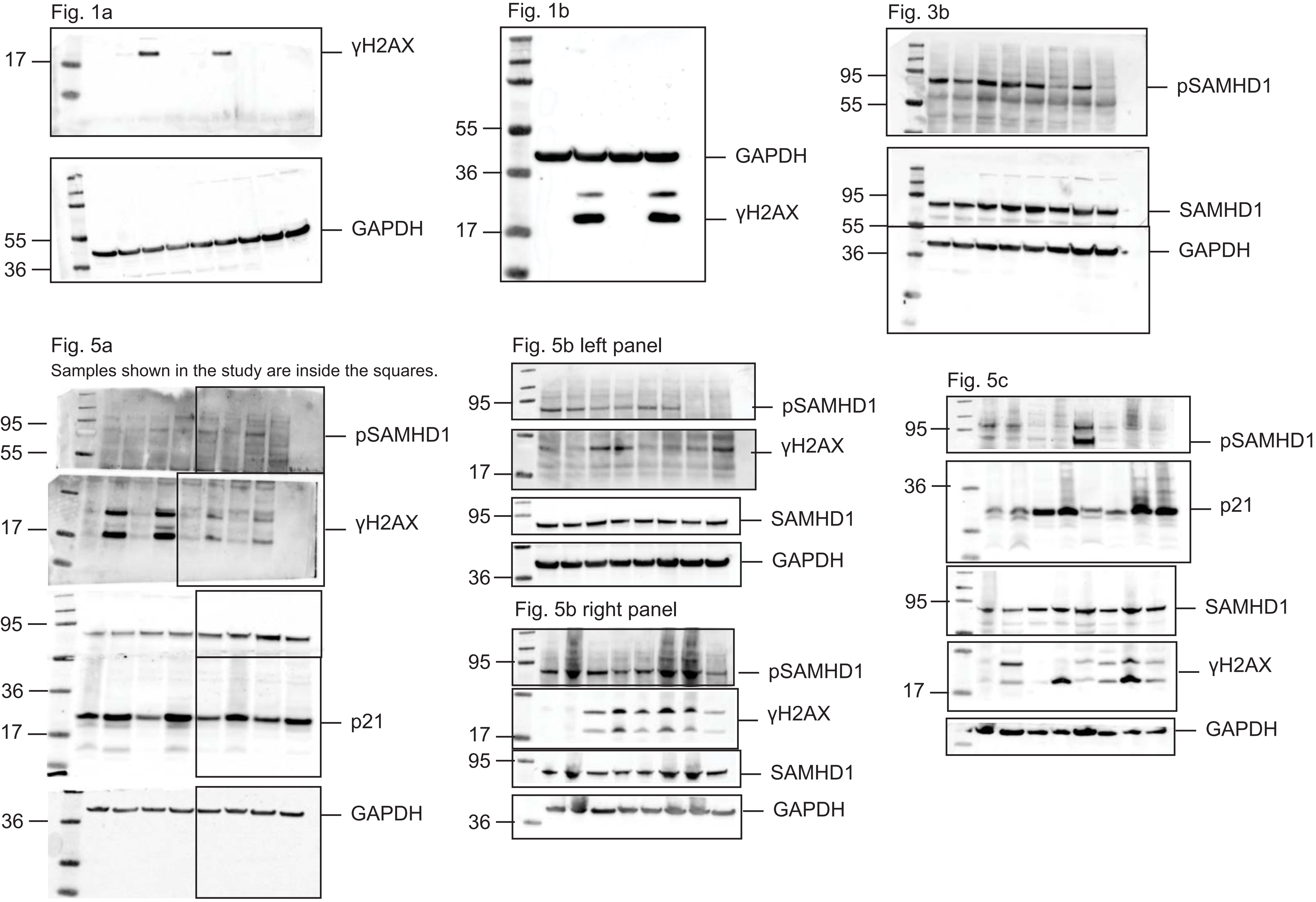

Supplementary figure 4. Original Uncropped Western Blots. Related to Figures 1, 3 and 5.

Fig. 5d Left panel

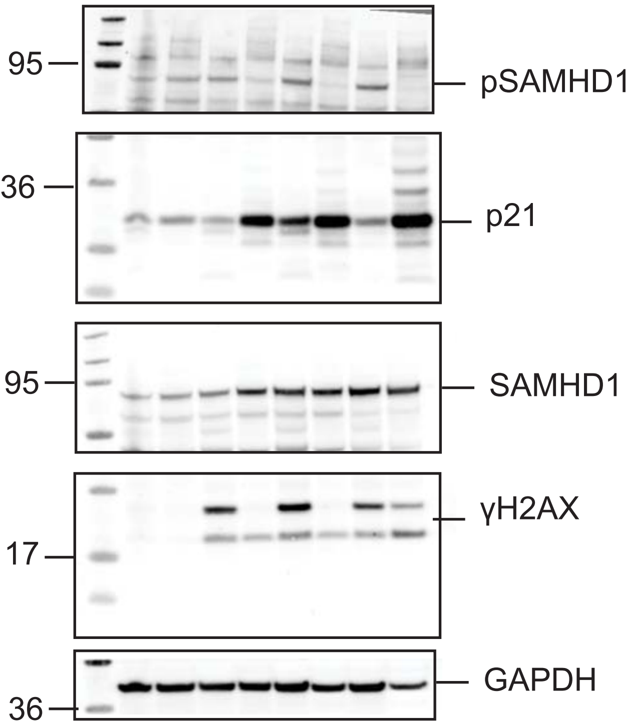

Fig. 5d Right panel

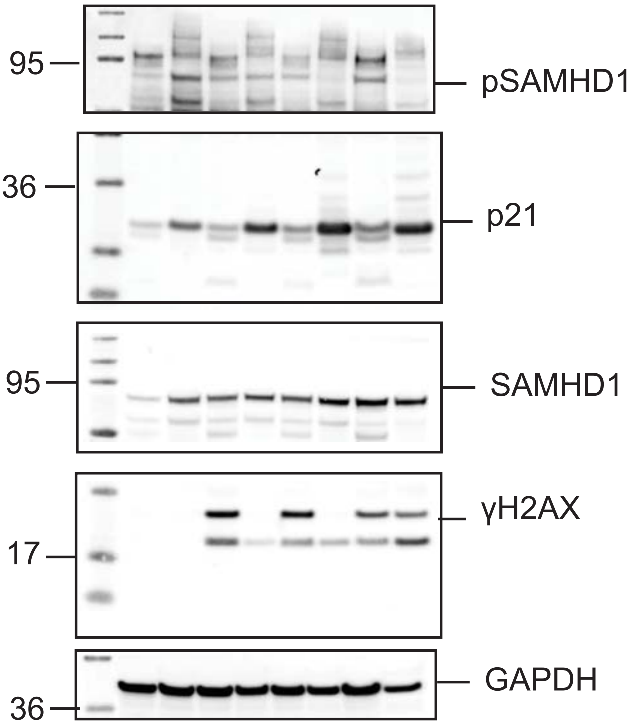

Fig. 5e Donor 337

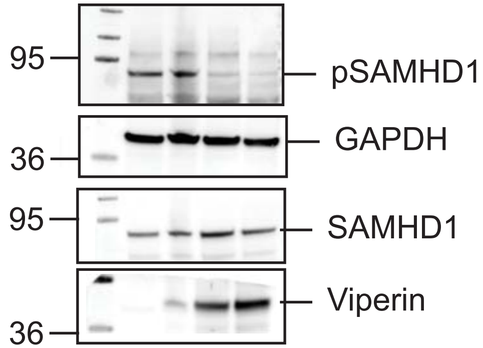

Fig. 5e Donor 338

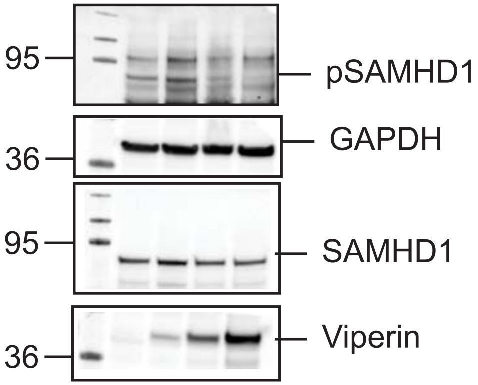

Supplementary figure 5. Original Uncropped Western Blots. Related to Figures 5.
